# Transcriptomic Insights Into the Growth Phase- and Sugar-Associated Changes in the Exopolysaccharide Production of a High EPS-Producing *Streptococcus thermophilus* ASCC 1275

**DOI:** 10.3389/fmicb.2018.01919

**Published:** 2018-08-20

**Authors:** Aparna Padmanabhan, Ying Tong, Qinglong Wu, Jiangwen Zhang, Nagendra P. Shah

**Affiliations:** ^1^Food and Nutritional Science, School of Biological Sciences, The University of Hong Kong, Pokfulam, Hong Kong; ^2^Cancer Genetics, School of Biological Sciences, The University of Hong Kong, Pokfulam, Hong Kong

**Keywords:** *S. thermophilus* ASCC 1275, exopolysaccharide, sugars, transcriptomics, RNA-seq

## Abstract

In a previous study, incorporation of high exopolysaccharide (EPS) producing dairy starter bacterium *Streptococcus thermophilus* ASCC 1275 was found to improve functionality of low fat mozzarella cheese and yogurt. This bacterium in its eps gene cluster has a unique pair of chain length determining genes, *epsC- epsD*, when compared to other sequenced *S. thermophilus* strains. Hence, the aim of this study was to understand the regulatory mechanism of EPS production in this bacterium using transcriptomic analysis to provide opportunities to improve the yield of EPS. As sugars are considered as one of the major determinants of EPS production, after preliminary screening, we selected three sugars, glucose, sucrose and lactose to identify the EPS producing mechanism of this bacterium in M17 medium. Complete RNA-seq analysis was performed using Illumina HiSeq 2000 sequencing system on *S. thermophilus* 1275 grown in three different sugars at two-time points, 5 h (log phase) and 10 h (stationary phase) to recognize the genes involved in sugar uptake, UDP-sugar formation, EPS assembly and export of EPS outside the bacterial cell. *S. thermophilus* 1275 was found to produce high amount of EPS (∼430 mg/L) in sucrose (1%) supplemented M17 medium when compared to other two sugars. Differential gene expression analysis revealed the involvement of phosphoenolpyruvate phosphotransferase system (PEP-PTS) for glucose and sucrose uptake, and *lacS* gene for lactose uptake. The pathways for the formation of UDP-glucose and UDP-galactose were highly upregulated in all the three sugars. In the presence of sucrose, *eps1C1D2C2D* were found to be highly expressed which refers to high EPS production. Protein homology study suggested the presence of Wzx/Wzy-dependent EPS synthesis and transport pathway in this bacterium. KEGG pathway and COG functional enrichment analysis were also performed to support the result. This is the first report providing the transcriptomic insights into the EPS production mechanism of a common dairy bacterium, *S. thermophilus.*

## Introduction

Lactic acid bacteria (LAB) have a long history of use to produce fermented foods (Holzapfel and Wood, 2014). There are numerous reports on exopolysaccharide (EPS) producing dairy LAB, *Streptococccus thermophilus, Lactococcus lactis* subsp. *lactis, Lactobacillus delbureckii* subsp. *bulgaricus, Lactobacillus plantarum*, and *Lactobacillus acidophilus*, which are widely used in the traditional and modern fermented food production, mainly yogurt, kefir and cheese (Bhaskaracharya and Shah, 2000; Wu et al., 2014; Yang et al., 2014). These long chain polymers produced by LAB are known to impart better quality, and sensory attributes to fermented foods in which they are grown (Lynch et al., 2018). EPS from LAB is of great focus in agro - food industry due to the generally regarded as safe (GRAS) status and their various health promoting effects (Badel et al., 2011). However, commercial exploitation of EPS from LAB has been a concern due to its low yield when compared to the EPS from plant and algal origin (Monsan et al., 2001; Torino et al., 2015).

Among LAB, *Streptococcus thermophilus* is a common dairy starter bacterium used in the manufacture of fermented foods such as yogurt and cheese. In our previous study, *S. thermophilus* 1275 was found to produce high amount of EPS (∼1 g/L) in whey protein isolate (WPI) supplemented milk at controlled fermentation conditions (37°C, pH 5.5) (Zisu and Shah, 2003). *S. thermophilus* 1275 was fully sequenced in our laboratory and its EPS gene cluster was compared with that of other five fully sequenced *S. thermophilus* strains (Wu et al., 2014). The results from full genome sequence (Wu et al., 2014) showed the presence of a novel EPS gene cluster that contains two sets of chain length determining genes, *epsC* – *epsD*, in the genome of this bacterium. This suggests our earlier findings about the ability of *S. thermophilus* 1275 to produce capsular and ropy EPS (Zisu and Shah, 2003). We have also performed a transcriptomics analysis for *S. thermophilus* 1275 grown in milk under different growth conditions to find the key regulatory genes involved in the pathway (Wu and Shah, 2018). However, we were unable to obtain a better insight into the EPS production mechanism due to insignificant expression levels of various EPS producing genes at high EPS producing conditions (Wu and Shah, 2018). Hence, in this study we investigated the EPS production mechanism in *S. thermophilus* 1275 through a detailed RNA-seq analysis under the presence of sugars that can influence EPS production.

Sugars are one of the major determinants of EPS production especially when a large-scale production of EPS is considered (Boels et al., 2001; Welman and Maddox, 2003; Tang et al., 2012). To shortlist the sugars for this study, carbohydrate metabolic pathway of *S. thermophilus* 1275 in Kyoto Encyclopedia of Genes and Genomes (KEGG) database were analyzed. Three sugars, glucose, sucrose and lactose, were chosen to examine the EPS production in the model bacterium *S. thermophilus* 1275. Due to the significant changes in EPS production in presence of each sugars at different stages of growth, we chose two-time points, one representing log phase (5 h) and other representing stationary phase (10 h) for the transcriptomics study. Furthermore, a comprehensive RNA-seq analysis was performed to fully understand the EPS production mechanism of *S. thermophilus* 1275 involving the import of sugars, UDP-sugar formation, EPS assembly and export of EPS.

## Materials and Methods

### Bacterial Strain and Fermentation Conditions

The high EPS producing diary bacterium *S. thermophilus* ASCC 1275 used in this study was obtained from Dairy Innovation Australian Limited (Werribee, VIC, Australia). The bacterium was stored at -80°C in M17 broth, (BD Company, Franklin Lakes, NJ, United States) containing 20% (v/v) glycerol until use. Three sugars, glucose (G), sucrose (S), and lactose (L), were chosen for this study after a preliminary screening. Working stocks were prepared by activating *S. thermophilus* 1275 twice at 37°C for 18 h using 1% inoculum; first in routinely used M17 broth containing 0.5% lactose and then in M17 broth containing 1% glucose (M17-G), 1% lactose (M17-L) or 1% sucrose (M17-S) as the sole carbon source. The bacterium activated in each sugar was then transferred into a GLS 80^®^ stirred reactor (Duran Group, Mainz, Germany) containing 1 L M17 fermentation media supplemented with 1% of the sugar in which it was activated. This process was repeated for all the three sugars. Our previous study showed optimum condition for EPS production in milk as 40°C and pH 5.5 (Zisu and Shah, 2003). However, due to buffering action of M17 broth we were unable to adjust the pH of the medium. Hence, in this study pH uncontrolled fermentation was carried out at 37°C for 24 h. *S. thermophilus* 1275 reaches log phase and stationary phase at different stages in each sugar (**Supplementary Figure S1a**). For making the comparison of gene expression easier, we selected two constant time-points in all sugars, one likely representing log phase and other stationary phase. Thus, samples (10 mL) were collected for RNA extraction from all the three sugars at these two fixed time points, 5 h representing log phase and 10 h representing stationary phase. Bacterial samples (*n* = 6; G5h, G10h, S5h, S10h, L5h, and L10h) were collected by centrifugation (10,000 × *g* for 15 min), and pellets were stored at -80°C after discarding supernatant. The fermentation experiments using each sugar were performed in triplicates.

### Estimation of OD, pH, EPS, Sugar Utilization and Lactic Acid Production

Samples (3 mL) were withdrawn at every 6 h from 0 h to 24 h to check the growth pattern, pH profile, sugar utilization and lactic acid production of *S. thermophilus* 1275 in the presence of the selected sugars. Growth of *S. thermophilus* 1275 was analyzed by measuring the optical density (OD) at 600 nm using a spectrophotometer and pH was measured using a portable pH meter. EPS was extracted from 50 ml fermented samples and estimated using phenol sulphuric acid method according to Li and Shah (2014). For high performance liquid chromatography (HPLC) analysis, 1 mL aliquot was centrifuged (10,000 × *g*, 10 min), supernatant was collected, filtered (0.45 μm Acrodisc^®^ syringe filters) and diluted 10 times using 5 mM H_2_SO_4_. Sugar utilization and lactic acid formation were simultaneously quantified from 20 μL of sample injected into Shimadzu model LC-2010A (Schimadzu Corp., Japan) system equipped with HPX-87H anion exchange column (300 × 7.8 mm, 9 μm, Bio-Rad Laboratories Inc., Hercules, CA, United States). An isocratic elution was performed using 5 mM H_2_SO_4_ at a flow rate of 0.8 mL/min for 30 min and column temperature 65°C. Refractive index detector and UV-Vis detector (220 nm) connected in series were used to determine sugar and lactic acid, simultaneously. All the above-mentioned parameters, pH, OD, EPS, sugar utilization and lactic acid production, were analyzed for the two-time points (5 and 10 h) considered in this study.

### RNA Extraction

Total RNA was extracted from *S. thermophilus* ASCC 1275 grown at different conditions using Ambion RiboPure^TM^-Yeast kit following the manufacturer’s instructions. The bacterial cells (approximately 3 × 10^8^) were lysed using 750 μL of 0.5 mm ice cold Zirconia beads, 480 μL lysis buffer, 480 μL phenol: chloroform: isoamylalcohol (25:24:1) and 48 μL 10% SDS for 10 min on a vortex adapter. The lysate was centrifuged at 15000 × *g* for 5 min to separate the RNA containing aqueous phase. Binding buffer (1.9 mL) and 100% ethanol (1.2 mL) was added into the aqueous lysate and mixed well, which was then passed through glass fiber cartridge. Impurities were removed using wash solutions and RNA was trapped in the filter that was collected by adding a low ionic strength elution solution. RNA was air dried and dissolved in 25 μL DEPC treated water. DNase I treatment was also performed to remove the contaminating DNA as outlined by the supplier.

The purity of RNA was checked using NanoDropTM and RNA integrity number (RIN) was analyzed using Agilent 2100 Bioanalyzer (Agilent Technologies, Santa Clara, CA, United States). From the QC report, RIN value of all samples was found to be 10 which indicates less RNA degradation. Also, all samples were found to be free from protein and phenol contamination thus qualifying for RNA-seq analysis.

### Transcriptomics Analysis

Trancriptome library construction including rRNA depletion, RNA fragmentation, cDNA synthesis, ends repair, A-tailing, adapter ligation, polymerase chain reaction (PCR) and sequencing were performed at Beijing Genomic Institute (Guangdong, China). rRNA depletion was carried out using Ribo-Zero Magnetic Kit for bacteria (Epicentre). The samples were then cleaned using RNAclean XP beads (Agencourt). Afterward, RNA was fragmented into 130–170 nt by adding fragmentation buffer (Ambion) into the samples and incubating at 70°C. The samples were again purified with RNAclean XP beads. The purified RNA was used for cDNA synthesis. The first cDNA strand was synthesized using First Strand Master Mix and Super Script II reverse transcriptase (Invitrogen). The mixture was incubated at 42°C for 50 min followed by inactivation at 70°C for 15 min. By using second strand Master mix, the second strand of cDNA was synthesized. Before PCR, end repair and poly A tail addition were performed using End pair repair Mix and A-tailing mix simultaneously. RNA index adapters were added to the adenylated 3′ end of DNA with the help of Ligation Mix. This DNA was used for PCR amplification with PCR Master mix and PCR primer cocktail for several rounds. The library obtained was validated using Agilent 2100 Bioanalyzer instrument (Agilent DNA 1000 Reagents) for determining the average molecule length and using real time PCR (q-PCR) for quantifying the library.

Sequencing of libraries was performed by amplifying on cBot to generate a cluster on the flow cell (TruSeq PE Cluster Kit V3-cBot-HS, Illumina, San Diego, CA, United States). The amplified flow cell was pair end sequenced on HiSeq 2000 System (TruSeq SBS KIT-HS V3, Illumina) to obtain read length of 90.

### Data Processing and Analysis

The raw reads stored in FASTAQ format were filtered prior to alignment by removing low quality sequences (quality threshold 20), bases and PCR duplicates to obtain clean reads. Indexing of clean reads to the reference genome of *S. thermophilus* 1275 (GenBank accession no. GCA_000698885.1) was performed using Bowtie 2 (Langmead and Salzberg, 2012) before mapping. Spliced aligner TopHat (Kim et al., 2013) 2.1.1 in very sensitive mode with only 2 bp mismatch allowed. TopHat maps paired cDNA fragments to the genome. Cufflinks 2.2.1 (Trapnell et al., 2012) was used for transcript assembly and to identify the differential gene expression (DEGs) in RNA-seq data. Six comparison groups (G5h vs. G10h, S5h vs. S10h, L5 vs. L10h, G10h vs. S10h and L10h, S10h Vs G10h and L10h, L10 Vs G10 and S10) were chosen from 6 samples to determine the DEGs. The normalization of the count of the reads generated corresponding to each gene was performed by calculating the reads per kilobase of transcript per million mapped fragments (RPKM) (Mortazavi et al., 2008). The gene IDs were annotated with the KEGG the gene annotation data. The *p*-value cutoff was kept as 0.05 and fold change cutoff as 1.5. The up regulated/downregulated genes were detected between 6 sample group. Functional analysis was conducted on the Kyoto Encyclopedia of Genes and Genomes (KEGG) pathway (Kanehisa and Goto, 2000) by hypergeometric test to identify the significantly activated/repressed pathways (*P* < 0.01), Cluster of Orthologus groups (COG) (Tatusov et al., 2000) and Gene Ontology (GO) (Ashburner et al., 2000) pathway.

### DEGs Validation Using RT-qPCR Assay

The RNA-seq results were validated by performing the RT-qPCR using StepOnePlus^TM^ Real-Time PCR system (Applied Biosystem, Foster City, CA, United States). Total fifteen DEGs were selected that included 5 genes from each condition G5h vs. G10h, S5h vs. S10h, L5h vs. L10h (**Table 2**). The total RNA samples extracted as described before was treated with RNase-free DNase I to remove contaminating DNA. High-Capacity RNA-to-cDNA^TM^ Kit (Applied Biosystems) was used to synthesize cDNA. RT-qPCR assay for a final volume of 25 μL were performed as follows: 95°C for 5 min; 40 cycles at 95°C for 10 s; at 55°C for 30 s and 72°C for 20 s (Wu et al., 2015). The cycle threshold (CT) value was determined and the relative gene expression of each target gene was calculated using comparative critical threshold method (2^-ΔΔCt^) method (Livak and Schmittgen, 2001). The mean log2fold-change value of RNA-seq analysis and -ΔΔCt value of qPCR assay of selected genes were compared.

### Statistical Analysis

All the results are presented as mean ± standard deviation (SD) of three independent biological replicates. One-way analysis of variance (ANOVA) was performed to find statistical significance (*P* < 0.05) among groups using IBM SPSS Statistics 20.0. Hypergeometric test (R package) was used to perform functional analysis (*P* < 0.01).

## Results

### Growth, EPS Production, Sugar Utilization and Lactic Acid Formation

**FIGURE 1:**
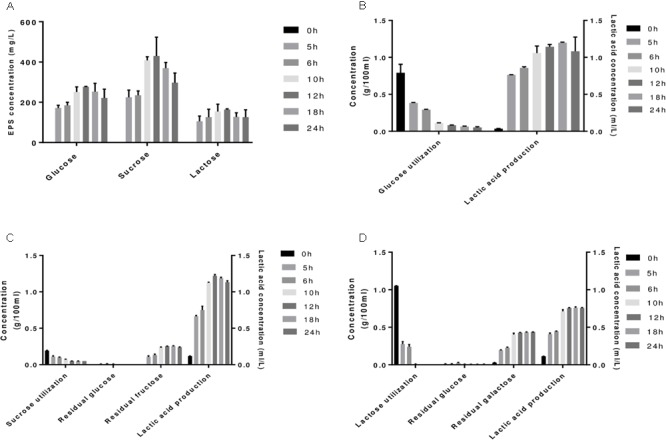
**(A)** EPS production of *S. thermophilus* 1275 in glucose (G-1%), sucrose (S-1%), lactose (L-1%) supplemented Ml7 media at different time points. Sugar utilization and lactic acid production in glucose **(B)**, sucrose **(C)** and lactose **(D)** media.

Sugars can modulate EPS synthesis in bacteria (Boels et al., 2001; Audy et al., 2010). In order to evaluate the potential sugars that can influence EPS production in *S. thermophilus* 1275, a preliminary screening was conducted using 5 sugars (glucose, sucrose, lactose, fructose, galactose) based on the information in the KEGG pathway of *S. thermophilus* 1275. The results showed that this bacterium can grow only in the presence of glucose, sucrose, and lactose. Growth pattern of *S. thermophilus* 1275 in M17 supplemented with 1% glucose, 1% sucrose or 1% lactose is shown in **Supplementary Figure S1a**. A rapid growth of *S. thermophilus* 1275 was observed in M17-G, attaining log phase in 2 h, when compared to sucrose (2.5 h) and lactose (4 h). The pH dropped gradually to 4.5 over time in M17-G and M17-S while pH remained at 5.5 from 12 h in M17-L (**Supplementary Figure S1b**). This is due to the unavailability of lactose in M17-L from 12 h onward as evident from the sugar utilization pattern (**Figure 1D**). Lactic acid production of 1.15 ml/L -1.2 ml/L was observed in M17-G and M17-S media at 12 h (**Figures 1B,C**). Furthermore, there was an accumulation of fructose and galactose in M17-S and M17-L media, respectively. EPS production was increased gradually in all the three sugars until 12 h and then dropped. Among the three sugars, sucrose produced maximum amount of EPS (∼430 mg/L) followed by glucose (∼276 mg/L) and lactose (∼163 mg/L) at 12 h (**Figure 1A**). It is apparent from these results that the pattern of growth and EPS production are markedly different in each sugar; this is a primary indication of differentially expressed genes (DEGs).

### Summary of Transcriptional Analysis and qPCR Validation

**FIGURE 2:**
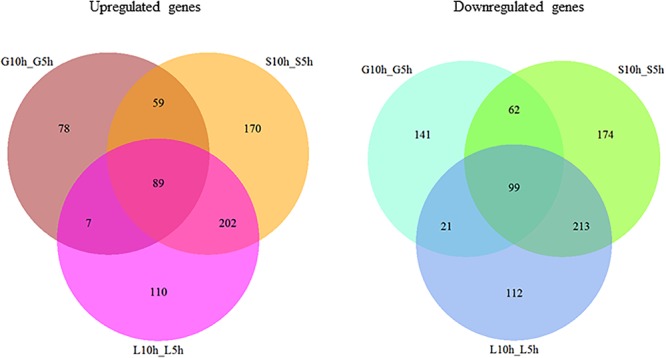
Venn diagram showing the number of differently expressed genes (>1.5 fold change, *P* < 0.01) during growth in M17 media supplemented with different sugars (glucose, sucrose, lactose) at logarithmic growth phase (5 h) and stationary growth phase (10 h).

**FIGURE 3:**
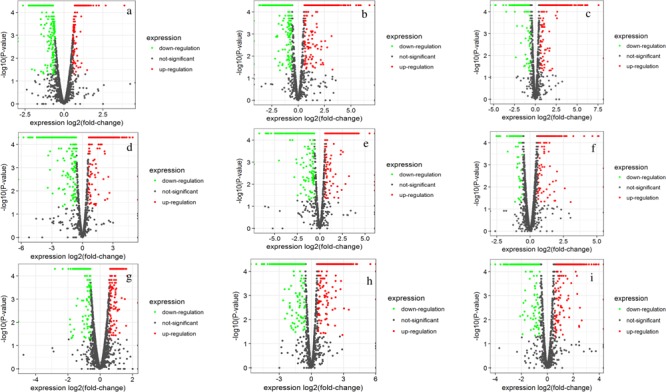
Volcano plots showing DEGs of *S*. *thermophilus* 1275 at G10h vs. G5h **(a)**, S10h vs. S5h **(b)**, L10h vs. L5h **(c)**, G5h vs. S5h **(d)**, G5h vs. L5h **(e)**, S5h vs. L5h **(f)**, G10h vs. S10h **(g)**, G10h vs. L10h **(h)**, S10h vs. L10h **(i).**

A whole genome transcriptome analysis was conducted to investigate variation in gene expression in presence of different sugars in *S. thermophilus* ASCC 1275. The primary goal of this study was to obtain insights into growth-associated and sugar-associated changes in the mRNA expression levels of *S. thermophilus* 1275 to get a better understanding about the EPS production in this bacterium. The cDNA library construction and sequencing of S. *thermophilus* 1275 at six different conditions generated 21,255,469 to 22,708,995 reads (**Table 1**). A minimum of 94.23% of total reads were mapped to the reference genome of ST1274 (GenBank accession no.: GCA_000698885.1). The reads mapped in proper pairs were more than 91% with Reads Per kilobases per Million reads (RPKM) values used to determined gene expression level showed that approximately 1600 genes were expressed in the 6 growth conditions of *S. thermophilus* 1275 (**Supplementary Table S1**). Overall, 233, 520 and 408 genes were up-regulated and 323, 548 and 445 genes were down-regulated, during growth associated changes in the media in presence of glucose, sucrose and lactose, respectively. The up/down regulated genes when compared among the glucose and sucrose were 148/161, sucrose and lactose were 291/312, glucose and lactose were 96/120 (**Figure 2** and **Supplementary Table S2**). Furthermore, 89 common DEGs were up-regulated and 99 common DEGs were down-regulated when compared among three sugars (**Figure 2** and **Supplementary Table S3**). Volcano plots revealed clear distinction in DEGs in different sugars at all the conditions (**Figure 3**).

**Table 1:** Summary of RNA-seq analysis.

Sample names	Total reads	Total mapped	Multiple mapped	Uniquely mapped	Read 1 mapped	Read 2 mapped	Reads mapped in proper pairs	Genes expressed
G5h	21,255,469 (100%)	20,392,675 (95.8%)	106,652 (0.49%)	20,286,024 (95.46%)	20,615,945 (96.99%)	20,169,405 (94.89%)	19,970,028 (93.96%)	1646
G10h	22,585,031 (100%)	21,264,264 (94.23%)	61,615 (0.27%)	20,862,426 (92.51%)	21,270,294 (94.18%)	21,258,235 (94.12%)	20,602,239 (91.34%)	1637
S5h	22,708,995 (100%)	21,787,704 (95.90%)	45,721 (0.20%)	21,741,983 (95.70%)	21,326,285 (93.87%)	22,042,456 (97.06%)	21,532,952 (94.82%)	1659
S10h	21,320,230 (100%)	20,398,893 (95.67%)	71,733 (0.34%)	20,327,160 (95.33%)	20,610,036 (96.67%)	20,187,749 (94.69%)	19,973,677 (93.67%)	1640
L5h	21,866,438 (100%)	20,936,036 (95.75%)	50,240 (0.23%)	20,885,796 (95.52%)	21,164,618 (96.79%)	20,707,454 (94.70%)	20,502,793 (93.76%)	1665
L10h	21,962,586 (100%)	20,797,946 (94.62%)	87,671 (0.40%)	20,710,275 (94.22%)	20,869,441 (95.02%)	20,726,452 (94.37%)	20,245,637 (92.04%)	1649

The validation of RNA-seq analysis was performed using qPCR assay of selected genes. The genes selected from different conditions were involved in the EPS biosynthesis pathway. For all the 15 selected genes, qPCR data and RNA seq data had accordance (**Table 2**). A good correlation was observed for the all the genes, suggesting RNA-seq data is valid.

**Table 2:** Primers designed for qPCR validation of RNA-seq data.

Group	Gene ID	Gene name	Amplicon size (bp)	Forward primer (5′→3′)	Reverse primer (3′→5′)	RNA-seq^a^	qPCR^b^
Reference gene		tuf gene	140	TAACGTCGGTGTCCTTCT	GACGTCCACCTTCTTCTTTAG		
G5h Vs G10h	T303_02795	PTS mannose transporter subunit IID	137	TTCTTCAACACTCACCCTTAC	GCAAGTGGACCCATCATAC	–1.38	–1.13
	T303_02800	PTS mannose transporter subunit IIC	171	GCTGAAGGTATCGGTGTTG	AAGGAGAGCGAGGTAGTATG	–0.74	–0.58
	T303_07665	PTS mannose transporter subunit IIAB	155	GGAGATGGGCTATATCAATCAG	AGTGACCAGCTGTGTATTTC	–0.49	–0.37
	T303_00105	UDP-glucose pyrophosphorylase	162	GCTGTTCTCCAAGCCAAA	CACAGGCATAACCGCAATA	0.59	0.41
	T303_06690	UDP-galactose-4-epimerase	144	CTTGGCTGCCGTCAAATA	CGCCCTTGAATACGGTAAG	1.37	1.19
S5h vs. S10h	T303_07880	UDP-glucose 4-epimerase	154	TTCACTTTGCGGCCTATTC	CCGTAAGTTGCTGCTGTT	2.89	2.30
	T303_07885	Galactose-1-phosphate uridylyltransferase	153	GCGGTAGCTTCTGATTATCC	GTAGGCATAAGGCGAGTATTG	3.36	2.93
	T303_07890	Galactokinase	169	GGGAACAAGAAGGAGTATTAGG	CCAGTTACTTCGGCAATATAGA	3.74	3.18
	T303_06336	epsN	149	CCTGCCTCCTTTCATCATC	CAACTCCAAGCTCTACTTCTAC	–1.67	–1.39
	T303_06885	Phosphoglycerate mutase	162	GTACTCAGCACACAAAGACC	CCATGTGCACCTACGAATAC	–1.37	–1.25
L5h vs. L10h	T303_07870	Lactose/galactose permease	155	CTTCAGGTAGCATGGGTAAAG	GATGCCAACGTGGATAAGAA	2.01	2.21
	T303_03155	6-phosphofructokinase	124	GACAAGGTAGAGATTGGTGAAG	TGAAGCCCGTAGCAGTAT	3.39	2.96
	T303_06085	4-alpha-glucanotransferase	156	CGTCAAGGTGAACCAATCA	CATACGCCATCTCCAGTTTC	3.90	3.37
	T303_08070	Peptide ABC transporter ATP-binding protein	162	CTGCGGCTAGTTTGAATGA	CGTGAGGGTAACGTGTTAAG	–0.73	–0.58
	T303_08085	Peptide ABC transporter permease	158	CAGCTACCAAGAGTCGTTATC	GACACCTGTTGACACACTATC	–1.22	–0.96

*^*a*^Mean log2fold change value of triplicates, ^*b*^-ΔΔCt value of qPCR analysis.*

### Sugar and Growth Associated Changes in EPS Bio-synthesis Associated Genes

Exopolysaccharide biosynthesis generally involve four steps: (1) transport of sugar into cytoplasm, (2) sugar-1-phosphate synthesis, (3) polymerization of EPS, and (4) export of EPS outside the bacterial cell (Laws et al., 2001). The DEGs involved in each step were analyzed to find the mechanism of EPS production in *S. thermophilus* 1275.

#### Transport of Sugar Into Cytoplasm

**FIGURE 4:**
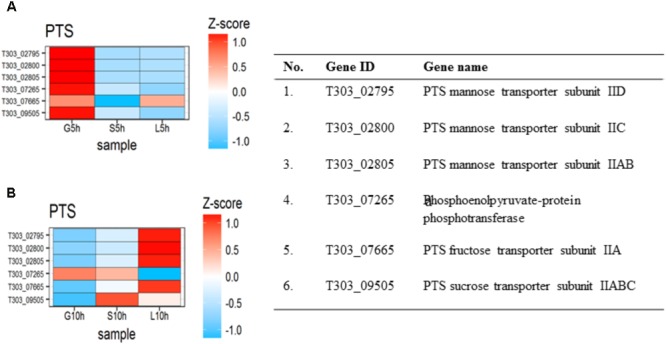
The DEGs in PTS transport system under die influence of different sugars at the two-time points **(A)** Heat map of DEGs involved in sugar transport at 5 h. **(B)** Heat map of DEGs involved in sugar transport at 10 h.

The effect of sugar transport system in the presence of glucose, sucrose and lactose are presented in **Figure 4**. In M17-G at 5 h, PTS mannose subunit IID (T303_02795), IIC (T303_02800), IIAB (T303_02805), PTS fructose transporter subunit IIA (T303_07665) and PTS sucrose transporter subunit IIABC (T303_09505) were highly expressed along with phosphoenolpyruvate-protein phosphotransferase (T303_07265); but at 10 h all other genes except phospho-enolpyruvate-protein phosphotransferase were downregulated. In M17-S at 5 h, we observed a downregulation of all the PTS gene; while at 10 h, PTS sucrose transporter subunit IIABC and phosphoenolpyruvate-protein phosphotransferase were upregulated. M17-L at 5 h showed only the upregulation of PTS fructose transporter subunit IIA, while at 10 h there was an upregulation of all the genes except phosphoenolpyruvate-protein phosphotransferase, which was highly downregulated. A two-fold increase in lactose permease (*lacS*) gene was also observe in M17-L at 10 h when compared to 5 h.

#### Amino Sugar and Nucleotide Sugar Metabolism

**FIGURE 5:**
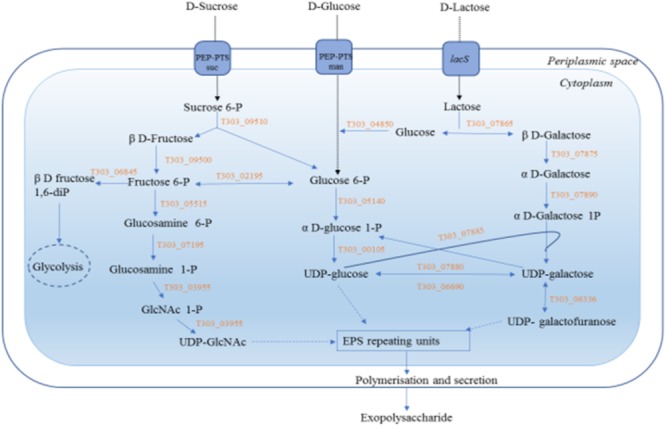
EPS biosynthesis pathway of *S. thermophilus 12 75* in the presence of three selected sugars, glucose, sucrose and lactose. Gene annotations:T303_09510 p-fructofuranosidase, T303_07865 *p*-galactosidase, T303_04850 glucokinase. T303_05140 phosphoglucomutase, T303_00105 UDP-glucose pyrophosphorylase, T303_07880 UDP-glucose 4-epinierase, T303_06690 UDP-galactose-4-epimerase, T303_07885 Galactose-1-phosphate uridylyltransferase, T303_07875 Galactose mutarotase, T303_07890 Galactokinase, T303_09500 Fructokinase, T303_06845 6-phosphofructokinase, T303_02195 Phosphoglucoseisomerase, T303_05515 glucosamine-fructose-6-phosphate aminotransferase, T303_07195 Phosphoglucosamine mutase, T303_03955 *N*-acetylglucosamine-1-phosphate uridyltransferase (bifunctional), T303_06336 UDP-galactopyranose mutase.

The expression of genes involved in amino and nucleotide sugar metabolism in *S. thermophilus* under the influence of three selected sugars are shown in **Figure 5**. In M17-G at 5 h, the genes responsible for the formation of glucose-1-phosphate (Glu-1P) phosphoglucomutase (T303_05140), glycogen phosphorylase (T303_06080) and glycogen debranching proteins (T303_07940, T303_07950) were found to be significantly up-regulated. One of the genes in Leloir pathway (T303_07880) that is responsible for UPD-glucose and UDP-galactose interconversion was found to be upregulated but the genes involved in the initial steps were found to be downregulated. At G10h, mannose transport was found to be significantly downregulated along with reduced expression of the genes responsible for nucleotide sugar synthesis.

In S5h, UDP-glucose pyrophosphorylase gene (T303_00105) that results in the formation of UDP-glucose and UDP-galactopyranose mutase gene (T303_06336) that results in the formation of UDP-galactofuranose were upregulated, along with the genes for β-galactosidase (T303_07865), phosphoglucoisomerase (T303_02195) and *N*-acetylglucosamine-1-phosphate uridyltransferase (T303_03955). At 10 h also the genes responsible for UDP glucose formation (UDP-glucose pyrophosphorylase, T303_00105; UDP galactose 4-epimerase, T303_06690) were found to be active. In case of lactose, at both the time points, all genes involved in Leloir pathway that lead to the formation of UDP-galactose and UDP-glucose; genes involved in the formation of UDP-*N*-acetylglucosamine and the gene UDP-galactopyranose mutase that lead to the formation of UDP-galactofuranose were found to be highly up-regulated. However, at 10 h there was a flux shift to glycolysis by the phosphoglucomutase (T303_05140), phosphoglucose isomerase (T303_02195) and 6-phosphofructokinase (T303_03155).

#### EPS Assembly

**FIGURE 6:**
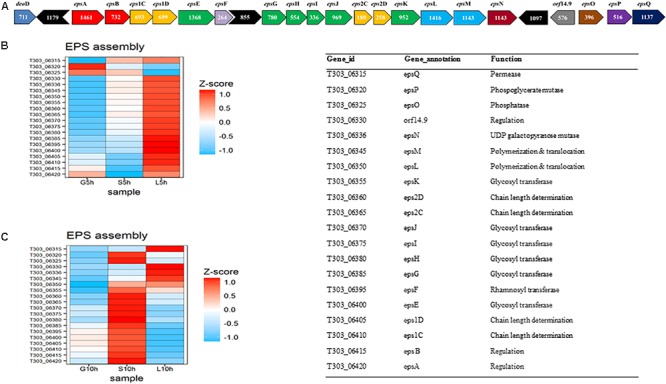
Gene expression pattern of EPS assembly genes, **(A)** EPS gene cluster of *S. thermophilus* 1275 (Wu et al., 2014). **(B)** Heat map of DEGs in EPS gene cluster at 5 h in the presence of glucose, sucrose and lactose, **(C)** Heat map of DEGs in EPS gene cluster at 10 h in the presence of glucose, sucrose, and lactose.

The investigation of differently expressed genes in EPS gene cluster under influence of different sugars, glucose, sucrose and lactose at the logarithmic- (5 h) and stationary growth phase (10 h) were performed (**Figure 6**). It was observed that in M17-lactose medium, all genes, except phosphorylating genes (*epsO* and *epsP*), were up-regulated at 5 h. The highly activated genes were the ones responsible for glycosidic linkage formation (*eps EFGHIJK*), chain length determination (*eps 2D2C*), polymerization, and translocation (*eps M L*) and secretion (*epsQ*). However, only 4 genes in the eps gene cluster *orf 14.9, eps Q, eps M* and *epsN* were expressed at 10 h in M17-L. In the case of M17-S, there was an upregulation of chain length determining genes (*eps2C2D*), glycosidic linkage formation genes (*eps FGHIJK*), polymerization and translocation gene (*epsML*) and secretion gene (*epsQ*) at 5 h. The expression of these genes was lower when compared to that of M17-lactose at 5 h. Interestingly, there was an overexpression of *epsAB1C1DOP* genes at 10 h in M17-sucrose medium when compared to other sugars, along with a slight upregulation in *epsFG* genes. Contrary to the other sugars, M17-glucose at 5 h showed up-regulation of only phosphorylating genes (*eps PO*) and regulatory gene (*epsA*); while, at 10 h there was an upregulation in the regulatory gene (*eps B*) as well as in the chain length determining genes (*eps 1C1D*).

#### Secretion of EPS

The secretion of EPS from cytoplasm to external environment can occur through Wzx/Wzy-dependent pathway, ABC transporter-dependent pathway, synthase-dependent pathway or by a sucrase protein (Schmid et al., 2015). In *S. thermophilus* 1275, we observed the presence of oligosaccharide repeat unit polymerase gene – *epsI* (T303_06375) and flippase gene – *epsLM* (T303_06350, T303_06345) in the *eps* gene cluster. This indicates the involvement of wzx/wzy dependent pathway (Reeves et al., 1996; Zeidan et al., 2017) in this bacterium. *epsL* and *epsI* was highly upregulated at 10 h in M17-S while *epsI* was highly downregulated in M17-L at 10 h. However, *epsM* was active at both time points in M17-L. Overall, in glucose medium, these genes were downregulated at the two-time points in this study.

### Carbohydrate Metabolism

**FIGURE 7:**
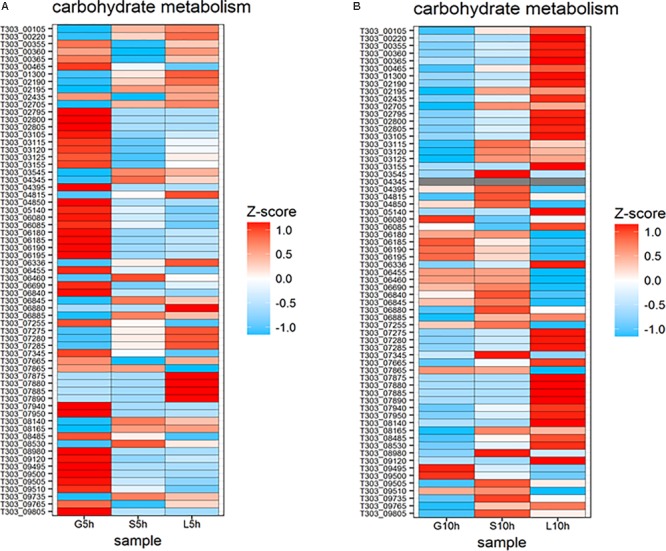
Changes in expression of genes associated with nucleotide sugar synthesis and carbohydrate metabolism. **(A)** Heat map of DEGs involved in nucleotide sugar synthesis and carbohydrate metabolism at 5 h in the presence of glucose, sucrose, and lactose. **(B)** Heat map of DEGs involved in nucleotide sugar synthesis and carbohydrate metabolism at 10 h in the presence of glucose, sucrose, and lactose.

The expression of genes involved in carbohydrate metabolic pathway were different in each sugar (**Figure 7**). Genes involved in glycolytic pathways (T303_00465, T303_04395, T303_04850, T303_05140, T303_06840, T303_07255, T303_07345, T303_08980, T303_09765) and pyruvate metabolism (T303_02435, T303_03105, T303_03115, T303_03120, T303_06180, T303_06185, T303_06190, T303_06195, T303_08485, T303_09120) were highly expressed at 5 h in M17-G but the expression of most of those genes were reduced or downregulated at 10 h. In sucrose medium at 5 h, only a few genes in the carbohydrate metabolism were up regulated including genes in pentose phosphate pathway (T303_01300, T303_02705, T303_06460), pyruvate metabolism (T303_00220, T303_06880, T303_08140, T303_08165) and some genes of glycolysis pathway (T303_02195, T303_03545, T303_04345, T303_06845, T303_09745). S10h showed the upregulation of more genes in carbohydrate metabolic pathway especially those responsible for glycolysis, pyruvate metabolism and pentose phosphate pathway. Genes involved in galactose metabolism (T303_07875, T303_7880, T303_07885, T303_07890) was found to be highly expressed at 5h in M17-L. In L10h, genes involved in citrate cycle (T303_07275, T303_07280, T303_07285), trehalose metabolism (T303_00355, T303_00360, T303_00365) and acetyl CoA carboxylase biotin carrier protein and subunits (T303_03105 T303_03115, T303_03120, T303_03125) were significantly upregulated.

### Amino Acid Metabolism

**FIGURE 8:**
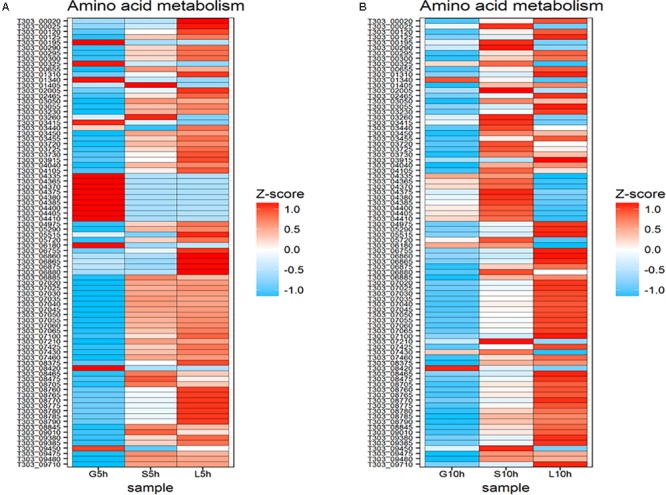
Changes in expression of genes associated with ammo acid metabolism. **(A)** Heat map of DEGs involved in amino acid metabolism at 5 h in the presence of glucose, sucrose and lactose. **(B)** Heat map of DEGs involved in amino acid metabolism at 10 h in the presence of glucose, sucrose, and lactose.

The changes in gene expression involved in amino acid metabolism in *S. thermophilus* 1275 are presented in **Figure 8**. At 5 h, genes involved in phenyl alanine biosynthesis (T303_04365, T303_04370, T303_04375, T303_04380, T303_04385, T303_04400, T303_04405, T303_04410) were found to be significantly upregulated in M17-G, but these genes were downregulated in M17-S and M17-L. Sucrose and lactose supplemented media had higher number of upregulated genes in amino acid metabolism. The genes involved in histidine metabolism (T303_07020, T303_07025, T303_07030, T303_07035, T303_07040, T303_07045, T303_07050, T303_07055, T303_07060, T303_07065) were upregulated in S5h and L5h, while these genes were significantly downregulated in M17-G. Genes responsible for tryptophan synthesis (T303_08760, T303_08765, T303_08770, T303_08775 T303_08780, T303_08785, T303_08790), arginine biosynthesis (T303_00020, T303_00025) and valine/leucine/isoleucine biosynthesis (T303_06860, T303_06865, T303_06875, T303_06880) were significantly upregulated in L5h when compared to other two sugars.

Ten-hour time point exhibited the downregulation of most of the genes associated with amino acid metabolism in glucose medium. Phenylalanine synthesis associated genes were upregulated but the histidine metabolism associated genes were down regulated at S10h. In L10h, pyrimidine metabolism was downregulated but expression of genes involved in histidine metabolism was increased.

### COG Analysis

Cluster of orthologus groups functional annotation and enrichment analysis were performed on upregulated and downregulated DEGs of the 9 groups to further understand their putative function. The data is presented in **Supplementary Tables S4, S5** with significantly enriched categories shown in bold.

The upregulated DEGs significantly enriched at different conditions are as follows: Carbohydrate transport and metabolism[G] in S5h vs. S10h; Carbohydrate transport and metabolism [G], and energy production and conversion [C] in L5h vs. L10h; Translation, ribosomal structure and biogenesis [J], Amino acid transport and metabolism [E], Defense mechanisms [V], and Cell wall/membrane/envelope biogenesis [M] in G5h vs. S5h; Amino acid transport and metabolism [E], Translation, ribosomal structure and biogenesis [J], and Defense mechanisms [V] in G5h vs. L5h; Amino acid transport and metabolism [E], Secondary metabolites biosynthesis, transport, and catabolism [Q] in S5h vs. L5h; Amino acid transport and metabolism [E] and Cell wall/membrane/ envelope biogenesis [M] in G10h vs. S10h; Translation, ribosomal structure and biogenesis [J], Defense mechanisms [V], Energy production and conversion [C] in G10h vs. L10h.

Downregulated DEGs significantly enriched in different conditions were: Coenzyme transport and metabolism [H] in G5h vs. G10h; Amino acid transport and metabolism [E], and translation, ribosomal structure and biogenesis [J] in S5h vs. S10h; Amino acid transport and metabolism [E], translation, ribosomal structure and biogenesis [J], cell wall/membrane/envelope biogenesis [M] and coenzyme transport and metabolism [H] in L5h vs. L10h; Carbohydrate transport and metabolism [G], lipid transport and metabolism [I], Inorganic ion transport and metabolism [P] in G5h vs. S5h; Carbohydrate transport and metabolism [G], Energy production and conversion [C], Lipid transport and metabolism [I] in G5h vs. L5h; Nucleotide transport and metabolism [F], Energy production and conversion [C], Post-translational modification, protein turnover, and chaperones [O] in S5h vs. L5h; Nucleotide transport and metabolism [F], Energy production and conversion [C], Carbohydrate transport and metabolism [G] in G10h vs. S10h; Inorganic ion transport and metabolism [P] in G10h vs. L10h.

### KEGG Pathway Analysis

**FIGURE 9:**
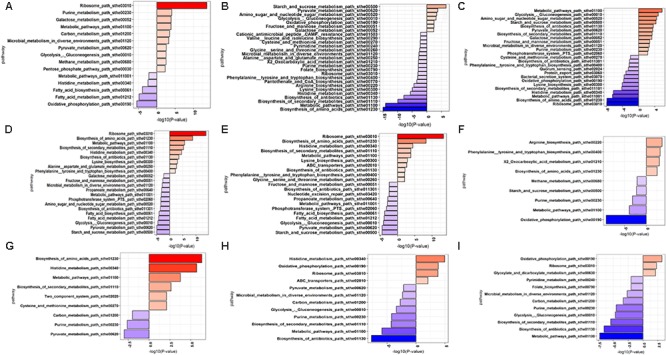
KEGG pathway enrichment analysis of DEGs 5h vs. G10h **(A)**, L5h vs. L10h **(B)**, S5h vs. S10h **(C)**, G5h vs. S5h **(D)**, G5h vs. L5h **(E)**, S5h vs. L5h **(F)**, G10h vs. S10h **(G)**, G10h vs. L10h **(H)**, S10h vs. L10h **(I)**.

Up-regulated and down-regulated DEGs were mapped to KEGG database and enrichment analysis were performed to identify the pathways of DEGs. The data is presented in **Figure 9** and **Supplementary Tables S6, S7**, with significantly enriched pathways in bold.

The pathways of amino sugar and nucleotide metabolism, pyruvate metabolism, fructose and mannose metabolism, microbial metabolism in diverse environment, purine metabolism, biotin metabolism, PTS system were found to be significantly expressed in high EPS producing sucrose supplemented medium. Biosynthesis of amino acids, metabolic pathways, purine and pyrimidine metabolism were significantly downregulated in lactose. Oxidative phosphorylation fatty acid metabolism, biotin metabolism and histidine metabolism were found to be downregulated in glucose supplemented medium at 10 h.

## Discussion

Exopolysaccharide produced by *S. thermophilus* is of interest in the food industry due to its ability to enhance the properties of fermented foods, like texture, mouthfeel, viscosity, and decrease the syneresis in yogurt (Purwandari et al., 2007). Low yield and complicated EPS production mechanism pose difficulty in the commercial exploitation of EPS from LAB (Welman and Maddox, 2003; Freitas et al., 2011). Hence, understanding the EPS production mechanism of potent EPS producers is of great importance in increasing the EPS yield. Various strategies like fermentation condition optimization and genetic manipulation have been used in improving EPS production in numerous LAB stains including *S. thermophilus* (Degeest et al., 1999; Zisu and Shah, 2003; Ruffing and Chen, 2006; Shene et al., 2008; Zhang et al., 2011; Papagianni, 2012). It is evident from previous work that one of the major contributors for EPS production is the carbohydrate or energy source used (Grobben et al., 1996; Degeest et al., 1999; Premjet et al., 2007; Suresh Kumar et al., 2007; Freitas et al., 2011). Thus, after preliminary screening, we chose glucose, sucrose and lactose to unravel insights into EPS production process in the organism of interest in our study, *S. thermophilus* 1275. The results showed that elevated amount of EPS (∼430 mg/L) was produced in sucrose (1%, w/v) supplemented medium when compared to that produced in glucose and lactose medium. Similarly, in the study of Zhang et al. (2011), another strain of *S. thermophilus* ST1 produced increased amount of EPS (135.8 mg/L) when 2% (w/v) sucrose and 0.5% (w/v) WPI were supplemented in skim milk under optimal conditions. However, another strain of *S. thermophilus* LY03 in the study of De Vuyst et al. (1998) produced high amount of EPS in milk medium/milk medium supplemented with lactose and protein sources.

In this study, we investigated EPS production mechanism in a high EPS producing dairy starter *S. thermophilus* 1275. Functional analysis based on genome-wide comparative transcriptomics was performed to understand the difference in cellular transcription that resulted in varied EPS production in *S. thermophilus* 1275. It was performed by altering the sugars, which are the sole carbon source, in the M17 broth in which they are grown. Three sugars, glucose, sucrose and lactose were selected after analyzing the KEGG database of *S. thermophilus* 1275. As fructose and galactose are one of the monomers present in sucrose and lactose, respectively, we also considered these two sugars while studying the sugar utilization of *S. thermophilus* 1275. However, it was observed that this bacterium was unable to utilize fructose and galactose (**Supplementary Figure S1**), and thus these sugars were eliminated from further study. The two-time points selected (5 and 10 h) gave a better understanding about the difference in gene expression in each sugar (**Supplementary Tables S1, S2**). Recently, an increasing number of researchers has focussed on incorporation of transcriptomics approach to understand the genetic mechanisms occurring in LAB. A growth-phase associated transcriptomics/proteomics study on probiotic *Lactobacillus rhamnosus* GG revealed some major changes during the transition from exponential to stationary phase: the shift of glycolysis to galactose utilization and expression of genes that promote survival of *L. rhamnosus* as well as those produce proteins which promote human health (Laakso et al., 2011). Another study on *L. rhamnosus* from dental pulp using next generation sequencing revealed the presence of a modified exopolysaccharide EPS gene cluster along with altered transcriptional regulators, ABC transporters for ferric ion and two-component sensor for kinase response regulator (Nadkarni et al., 2014). There was an indication of *eps* gene cluster downregulation in *L. rhamnosus* GG in the presence of bile (Koskenniemi et al., 2010). However, our work is the first study that directly focused on the comparative transcriptomic analysis approach to identify the EPS production mechanism under the influence of various sugars. Thus, our study introduces a new approach in understanding the EPS production.

### Investigation of *S. thermophilus* 1275 EPS Production Mechanism

#### Uptake of Sugars Into Cytoplasm

Sugar transport from the external growth media to cytoplasm can influence EPS production and it is a highly regulated process (Laws et al., 2001). Previous reports showed that sugar transfer in *S. thermophilus* involved an active transport system, either a phosphoenolpyruvate (PEP): sugar phosphotransferase system (PTS) dependent (group translocation system) or PEP-PTS independent (primary transport using ATP or secondary transport using electrochemical gradient) system (Freitas et al., 2011; Harutoshi, 2013; Cui et al., 2017). In *S. thermophilus* 1275, the upregulations of genes involved in PEP-PTS were observed (**Figure 4**) which indicate the involvement of PEP group translocation system for sugar transport.

Many streptococci and *Escherichia coli* are known to transport glucose using multi-enzymatic PTS mannose transporters (Vadeboncoeur and Pelletier, 1997; Cochu et al., 2003; Steinsiek and Bettenbrock, 2012). Similarly, in *S. thermophilus* 1275, glucose transport was performed using a functional PTS mannose transporter consisting of subunits IIAB, IIC and IID (**Figure 4**). For sucrose transport, *S. thermophilus* 1275 used a sucrose specific PTS system (Barrangou et al., 2006). Lactose uptake by *S. thermophilus* 1275 was performed mainly by lactose permease (*lacS*) gene (Van den Bogaard et al., 2004; Barrangou et al., 2006) as reported in our previous study (Wu and Shah, 2018). A two- fold increase in *lacS* expression was observed at 10 h. A slight upregulation in PTS fructose transporter IIA (*celB*) gene was also observed in M17-L. Even though, *celB* is putative transporter for cellobiose, a point mutation in the promoter increased lactose uptake in *Lactococcus lactis* MG1363 (Solopova et al., 2012). All the sugar transported by PEP-PTS system is phosphorylated to sugar 6-phosphates but *lacS* is a symport (Yeagle, 2016).

#### Nucleotide Sugar Synthesis

The sugar nucleotides and amino sugars are the precursors for EPS biosynthesis (Boels et al., 2001). The formation of sugar nucleotides solely depends on the type of sugar phosphates formed from the transported sugars. Sugar-1- phosphates can lead to EPS formation by the formation of sugar nucleotides; when sugar-6-phosphate enters Embden-Meyerhof-Parnas (EMP) pathway via the formation of fructose-6-phosphate (Cui et al., 2016). Understanding the genes responsible for increasing sugar nucleotide formation is essential as it can be engineered to increase the carbon flux toward polymer production (Schmid et al., 2015). Three sugars, glucose, sucrose and lactose, used in the study enters the cytoplasm of *S. thermophilus* 1275 through PEP-PTS transporters (**Figure 4**), and then form sugar-6-phosphates. Phosphoglucomutase (PGM) is a key enzyme which determines whether sugar-6P enter glycolysis or EPS biosynthesis pathway (Degeest and De Vuyst, 2000). PGM that converts glucose-6-phosphate (Gluc-6P) to glucose-1-phosphate (Gluc-1P) was found to be upregulated along with the downregulation of phosphoglucose isomerase (PGI) that leads Gluc-6P to glycolysis, at G5h. Along with PGM, other genes that lead to the production of Gluc-1P like glycogen phosphorylase and glycogen debranching proteins were also found to be up-regulated. This shift the flux toward nucleotide sugar synthesis and thus increase EPS production. The enzyme UDP galactose 4-epimerase (reversible conversion of UDP-galactose to UDP-glucose) which performs the final step of Leloir pathway was overexpressed at 5 h in M17-G and the enzymes in the initial steps were found to be downregulated (**Figure 7**). This indicate that formation of UDP-sugars for EPS synthesis starts at an earlier stage in glucose medium when compared to other sugars. It can be due to the rapid utilization of glucose by *S. thermophilus* 1275 and thus reaching late log phase at 5 h, unlike in sucrose and lactose medium (**Supplementary Figure S1a**).

At both time points in sucrose supplemented medium, genes responsible for the formation of UDP-glucose was mainly upregulated. In case of lactose, at both the time points, all genes involved in Leloir pathway that lead to the formation of UDP- galactose and UDP-glucose, UDP-N-acetylglucosamine and UDP-galactofuranose were found to be highly upregulated. However, at 10 h, there was a flux shift to glycolysis by the PGM, PGI and 6-PFK. It was observed that lactose was exhausted in the media after 6 h (**Figure 7**). Hence, to regain the energy for cellular mechanism in the bacterium the pathway was shifted to glycolysis which can be the reason for decrease in EPS production in lactose medium. (Ramos et al., 2001). In a previous study by Li and Shah (2016), the sugars mannose, galactose and glucose were identified as the major monomers of EPS produced by *S. thermophilus* 1275. However, no gene that lead to the production of GDP-mannose was detected in this study and in the KEGG database of *S. thermophilus* 1275. The report of Wu and Shah (2018) also support this finding. Previous studies on *L. paracasei* LC2W (Xu et al., 2015) and *L. kefiranofaciens* ZW3 (Xing et al., 2017) indicated the absence of corresponding genes that lead to GDP-mannose production in these strains, suggesting that the mannose in its EPS might be derived from medium. From this study we understand that UDP-glucose and UDP-galactose should be the major nucleotide sugars involved in EPS production by *S. thermophilus* 1275 with traces of UDP-*N*-acetylglucosamine and UDP-galactofuranose. Further purification and characterization of EPS must be performed to identify the structure and chemical composition of this EPS.

#### EPS Assembly

The synthesis and transport of EPS has been carried out by a set of functional genes called eps gene cluster. The diversity of EPS produced depends on the variations in the genetic make-up of this gene cluster (Cui et al., 2016). *S. thermophilus* 1275 is reported to have a distinct *eps* gene cluster in the chromosome with two sets of chain length determining genes *epsC* and *epsD* (Wu et al., 2014). Unique *eps* gene clusters are also identified in the study of Nadkarni et al. (2014) for *L. rhamnosus* clinical isolates (LRHMDP2 and LRHMDP3) and of Hao et al. (2011) for *L. bulgaricus* 2038 using comparative genomics. In our study, the expression of genes in eps gene cluster was distinct in each sugar at two-time points (**Figure 6**). The sucrose medium, which showed high EPS production had both sets of chain length determining genes, *eps 2C2D1C1D*, up-regulated at 10 h. In M17-L at 5 h, *eps 2C2D1C1D* were upregulated but at 10 h expression of these genes was reduced (**Figure 6**). This resulted in decreased EPS production in lactose when compared to sucrose at 10 h. In a previous study conducted in our laboratory, an increase in gene expression of *eps1C*-*eps 1D* was observed with an increase in temperature from 37 to 40°C in milk (pH 5.5) which resulted in an improved EPS production (Wu and Shah, 2018). In the current study, protein homology test using BLAST revealed that the gene product of *eps2C* and *eps2D* as polysaccharide biosynthesis proteins, *eps1C* as capsular biosynthesis protein *cpsC*, and *eps1D* as tyrosine protein kinase. Reports suggest that the modulatory proteins *epsC* (*cpsC* or *wzd*) and *epsD* (*cpsD* or *wze*) can likely be substitutes (Nourikyan et al., 2015; Grangeasse, 2016; Zeidan et al., 2017). UDP-galactopyranose mutase gene which is responsible for UDP-galactofuranose formation was downregulated over time in sucrose medium but the gene was active in lactose medium at both time points. The presence of this gene was also reported in *Streptococcus thermophilus* MN-BM-A01, however, this is considered as a rarely found gene in *S. thermophilus.*

Even though, there was a significant upregulation in most of the genes in eps gene cluster at 5 h in M17-lactose medium, the less amount of EPS production was due to the lack of lactose in the medium (**Figure 1C**) and downregulation of all the genes at 10 h. Contrary to the other sugars, M17-glucose at 5 h showed up-regulation of only phosphorylating genes and regulatory gene; while at 10 h there was a slight upregulation in the chain length determining genes (*eps 1C1D*).

#### EPS Secretion

In LAB, Wzx/Wzy-dependent exopolysaccharide production is mainly focused on *Streptococcus, Lactobacillus* and *Lactococcus* (Zeidan et al., 2017). Our protein homology study suggests the presence of Wzx/Wzy-dependent pathway in *S. thermophilus* 1275. The initial step of Wzx/Wzy- dependent pathway is the activation of undecaprenyl-phosphate, which happens at the inner membrane by an active sugar precursor using priming glycosyl transferase. Flippase translocates this across cytoplasmic membrane. Next, polymerization occurs using *wzy* polymerase at the periplasmic space and transported to the cell surface (Cuthbertson et al., 2009; Rehm, 2010; Islam and Lam, 2014; Schmid et al., 2015). The exopolysaccharides produced by Wzx/Wzy-dependent pathway are usually heteropolysaccharides (Schmid et al., 2015). The protein homology test using BLAST showed that the eps gene cluster of *S. thermophilus* 1275 has oligosaccharide repeat unit polymerase (*epsI*), O-unit flippase *wzx* (*epsM*) and flippase (*epsL*), which was upregulated in sucrose and lactose medium. However, further studies including knock out experiments and X-ray crystallographic studies must be performed to confirm *wzy* status for the polymerase gene (Islam and Lam, 2014) due to its low sequence identity (36%) to other *wzy* genes in the database. This least sequence similarity showed by *wzy* genes, even among same species, has already been reported (Islam and Lam, 2014). Also, the genes encoding similar functions are named differently even in same species. Polymerase and flippase are coded by *epsK* and *epsM* in *L. lactis* SMQ-461 (Dabour and LaPointe, 2005), and with *epsI* and *epsK* in *L. lactis* NIZO strain B40 (Kranenburg et al., 1997).

#### COG and KEGG Analysis

The pathways and functions of DEGs responsible for EPS production under the influence of glucose, sucrose and lactose in *S. thermophilus* 1275 were studied using enrichment analysis. Analysis of COG category enrichment showed that the significantly enriched up-regulated terms at high EPS producing condition in sucrose supplemented media at 10 h was “carbohydrate transport and metabolism.” When compared with glucose “translation, ribosomal structure and biogenesis,” “amino acid transport and metabolism,” “defense mechanisms,” “cell wall/membrane/envelope biogenesis were enriched at 5 h,” and “amino acid transport and metabolism,” “cell wall/membrane/envelope biogenesis” were enriched at 10 h. The enrichment of amino acid transport and metabolism was also observed in high EPS producing condition (WPI supplemented milk, pH 5.5, 37°C) in *S. thermophilus* 1275 (Wu and Shah, 2018). In lactose supplemented media “nucleotide transport and metabolism” was mainly enriched in both time points. A significant enrichment of “energy production and conversion” was observed in lactose supplemented media at 10 h when compared to glucose and sucrose. This support the flux shift to glycolysis observed before (**Figure 7**).

The KEGG enrichment analysis showed the significant enrichment of pathways involved in “amino sugar and nucleotide sugar metabolism” in both M17-S and M17-L at 10 h. However, there was a significant enrichment of “oxidative phosphorylation” in M17-L which indicate energy production. This again indicate the shift toward glycolytic pathway at 10 h in M17-L reducing the EPS production.

## Conclusion

A global insight into the EPS production mechanism of *S. thermophilus* 1275 under the influence of three major sugars, glucose, sucrose and lactose, was obtained after genome wide transcriptomics analysis. High amount of EPS was produced in sucrose-supplemented medium. PEP-PTS mediated sugar transport (glucose, sucrose) and *lacS* gene mediated transport (lactose) were observed for sugar transport. The upregulation of chain determining genes in the eps gene cluster was found to improve EPS production in *S. thermophilus* 1275. UDP-glucose and UDP-galactose synthesizing gene were mainly upregulated in all sugars. Wzx/Wzy pathway was suggested for EPS polymerization and transport. A well-co-ordinated regulation of eps gene cluster and carbohydrate metabolizing genes are required for high EPS production.

## Author Contributions

AP, QW, and NS conceived the research idea. AP designed and performed the experiments except mRNA sequencing. AP, YT, and JZ conducted bioinformatics analysis. AP analyzed and interpreted the results. AP and NS drafted the manuscript.

## Conflict of Interest Statement

The authors declare that the research was conducted in the absence of any commercial or financial relationships that could be construed as a potential conflict of interest.
