# Supplementary material for: Transcriptomic Insights Into the Growth Phase- and Sugar-Associated Changes in the Exopolysaccharide Production of a High EPS-Producing Streptococcus thermophilus ASCC 1275
Source: Front Microbiol. 2018 Aug 20;9:1919. doi: 10.3389/fmicb.2018.01919 (PMC6109772; doi:10.3389/fmicb.2018.01919)
Supplement: Supplementary file 8 [file Image_1.PDF]

Supplementary figures of

Transcriptomic insights into the growth phase- and sugar-associated changes in the exopolysaccharide production of a high EPS-producing *Streptococcus thermophilus* ASCC 1275

Aparna Padmanabhan[1], Ying Tong[2] , Qinglong Wu[1†], Jiangwen Zhang[2], Nagendra P. Shah[1*]

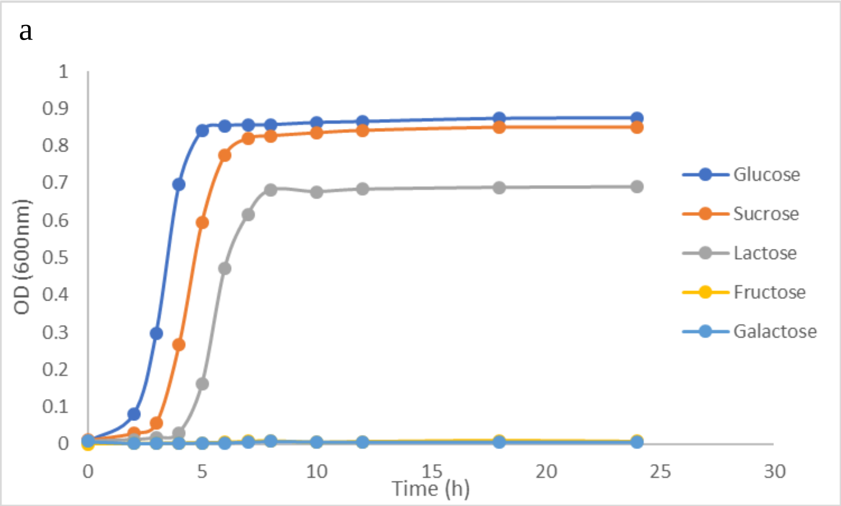

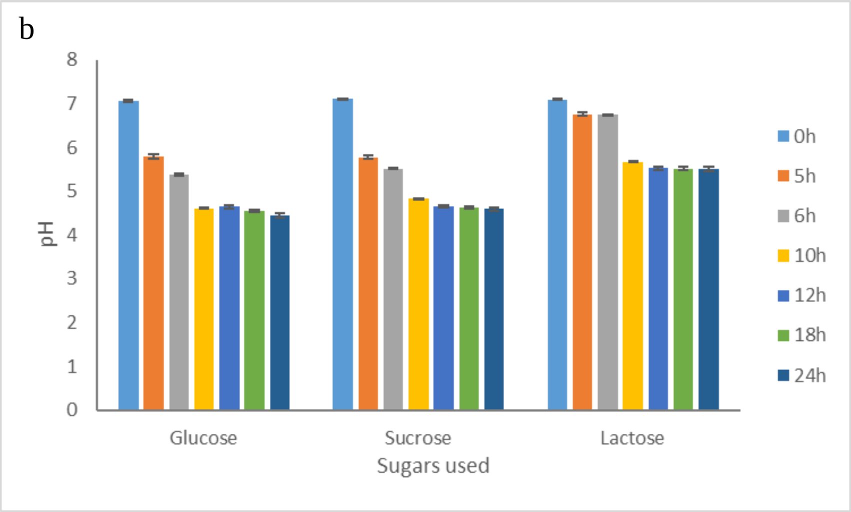

Fig S1. a) Growth curve of *Streptococcus thermophilus* 1275 under the influence of different sugars. b) pH changes over time of fermentation.
